# Regulation of ADAM10 by MicroRNA-23a Contributes to Epileptogenesis in Pilocarpine-Induced Status Epilepticus Mice

**DOI:** 10.3389/fncel.2019.00180

**Published:** 2019-04-30

**Authors:** Xinjian Zhu, Yuanyuan Yao, Yaoyao Liu, Rong Zhou, Wei Zhang, Qiang Hu, Hang Liu, Mohammed Hidayath Al Hamda, Aifeng Zhang

**Affiliations:** ^1^Department of Pharmacology, Medical School of Southeast University, Nanjing, China; ^2^Department of Pathology, Medical School of Southeast University, Nanjing, China

**Keywords:** ADAM10, microRNA-23a, hippocampus, status epilepticus, epileptogenesis

## Abstract

ADAM10, a member of the disintegrin and metalloproteinase domain-containing protein (ADAM) family, has been reported to mediate proteolytic shedding of cell surface proteins. An increasing body of evidence indicates that ADAM10 is involved in various neurological disorders including epilepsy. However, the molecular mechanisms underlying the regulation of ADAM10 expression in the epileptic brain remain poorly understood. In this study, we demonstrate that ADAM10 is targeted by microRNA-23a (miR-23a) in the hippocampus. Inhibition of miR-23a increased hippocampal ADAM10 expression while an increase in miR-23a suppressed hippocampal ADAM10 expression in pilocarpine-induced status epilepticus (SE) mice. Furthermore, inhibition of miR-23a suppressed spontaneous recurrent seizures through up-regulation of ADAM10 in pilocarpine-induced SE mice. Our findings suggest that miR-23a targeting of ADAM10 contributes to epileptogenesis in temporal lobe epilepsy. Thus, the miR-23a-ADAM10 pathway in the epileptic brain may provide a novel target for the treatment of epilepsy.

## Introduction

Temporal lobe epilepsy (TLE) is the most common type of epilepsy in adults, which is characterized by recurrent, unprovoked focal seizures that originate in the temporal lobe of the brain. Abnormalities in excitatory and inhibitory neurotransmission are considered to be the underlying cause of TLE. However, the molecular and cellular mechanisms underlying TLE remain unclear. ADAM10 is a member of the ADAM metalloprotease family, which is a key proteinase responsible for the processing of amyloid precursor protein (APP) to prevent the excessive production of the pathogenic amyloid β (Aβ) peptide (Postina et al., 2004; Kuhn et al., 2010), a hallmark of Alzheimer’s disease (AD). It has been reported that ADAM10 is largely distributed in the brains of both rodents and humans (Yavari et al., 1998; Karkkainen et al., 2000). ADAM10 mRNA was found to be expressed throughout the whole brain, including the olfactory bulb, hippocampus, and the subthalamic regions (Karkkainen et al., 2000). The presence of ADAM10 in the brain raises the possibility that it may have an important role in pathophysiological conditions of the central nervous system. In addition to the role of ADAM10 in AD, recent studies suggest that ADAM10 may contribute to other neurological diseases including fragile X syndrome, traumatic brain injury, and psychiatric disorders (Warren et al., 2012; Pasciuto et al., 2015; Zilhao et al., 2015; Del Turco et al., 2016). A growing body of evidence suggests that ADAM10 may also play an important role in epilepsy. A previous study reported that ADAM10 expression is significantly altered in the dentate gyrus (DG) of the hippocampus after a kainic acid-induced generalized seizure (Ortiz et al., 2005). Impairment in APP processing in the absence of ADAM10 may lead to increased network hyperexcitability and seizures in the brain (Prox et al., 2013). Conditional ADAM10 knockout causes axon-mistargeting and neuronal network dysfunction, which may contribute to epileptogenesis (Kuhn et al., 2016).

MicroRNAs (miRNAs) are a family of small (19∼24 nucleotides) non-coding RNAs (ncRNAs) that post-transcriptionally modulate gene expression by mediating either mRNA degradation or translation repression (Bartel, 2004). An increasing body of evidence has indicated that miRNAs are key mediators in both physiological process and pathological conditions. In epilepsy, miRNAs have a distinct expression pattern and modulate expression of specific genes during pathological development (Tan et al., 2013; Gorter et al., 2014; Henshall, 2014; Noebels, 2015). Recent studies have demonstrated that miR-23a expression is significantly altered in the epileptic brain of both human and animal models (Song et al., 2011; Henshall, 2014; Roncon et al., 2015). In particular, Song et al. (2011), reported that miR-23a expression in the hippocampus of TLE rats was predominantly up-regulated in the acute phase with the peak at 24 h post-status epilepticus (SE). In latent period, it was down-regulated with the valley at 10 days post-SE. In chronic epileptic phase it was up-regulated again with the peak at 50 days post-SE. Consistently, Roncon et al. (2015), reported that miR-23a was up-regulated in the brains of both epileptic humans and rats in chronic epilepsy period, suggesting that miR-23a may play an important role in the development of epilepsy by targeting genes related to epilepsy. In the present study, we sought to determine whether miR-23a regulates epileptogenesis through targeting the ADAM10 gene in TLE mice.

## Materials and Methods

### Animals

Male C57BL/6J mice (4–6 weeks old; weighing 19 ± 2 g at the beginning of the experiments) were obtained from Nanjing Biomedical Research Institute of Nanjing University (NBRI) (Nanjing, China). The animals were housed in plastic cages and kept in a regulated environment (22 ± 1°C) with an artificial 12 h light/dark cycle (lighted from 7:00 A.M. to 7:00 P.M.). Food and tap water were available *ad libitum*. Procedures for pilocarpine-induced SE model and all subsequent experiments were approved by the Animal Care and Use Committee of Medical School of Southeast University. All efforts were made to minimize animal suffering and discomfort and to reduce the number of animals used.

### Surgery and Virus Injection

For lentiviral infection, the mice were anesthetized and positioned on a stereotaxic frame (Stoelting, Wood Dale, IL, United States). Vectors (lentiviral shRNA-ADAM10 or lentiviral shRNA-Ctrl) were bilaterally injected into the hippocampus (Coordinates: A/P −2.2; M/L ± 2.0; D/V 1.9) using 1 μl of viral preparation at a rate of 0.2 μl/min. ADAM10-shRNA lentiviral particles and control lentiviral particles were purchased from Santa Cruz Biotechnology Inc. (Santa Cruz, TX, United States). For EEG recording, mice were anaesthetized and placed in a stereotaxic frame for hippocampus depth electrode placement as we previously described (Zhu et al., 2012). A bipolar twist electrode was placed in the left hippocampus (Coordinates: A/P −2.2; M/L −2.0; D/V 1.9) for continuous EEG monitoring. In addition to the hippocampal electrodes, four cortical screws with two in front of bregma for bilateral cortex recording, and two behind lambda for ground and reference. Electrodes connected with a plastic cap and kept in place with dental cement.

### *In vivo* Drug Treatments

Up- or down-regulation of miR-23a expression in the hippocampus of TLE mice was achieved by intracerebroventricular (i.c.v.) delivering of synthetic miR-23a agomir or antagomir into the right lateral ventricle as we previously described (Zhu et al., 2017). Briefly, mice were anesthetized and positioned in a stereotaxic instrument with a mouse adapter (David Kopf Instruments, Tujunga, CA, United States). The stereotaxic coordinates for implantation of guide cannula into right lateral ventricle were according to the mouse brain atlas (AP = 0.5 mm relative to bregama; ML = 0.8 mm; DV = −2.5 mm from the skull surface). The guide cannula was then affixed with dental cement. All injections were 1 μl and the injections were carried out over 60 s and the syringe was left in place for additional 2 min to minimize backflow after each injection. The miR-23a agomir, antagomir, and scrambled RNA oligonucleotides negative control were obtained from Sangon Corporation (Sangon Biotech).

### Pilocarpine Induction of SE and Video EEG Recording

Status epilepticus model was induced as we previously described (Zhu et al., 2012). Briefly, mice were subject to an intraperitoneal injection of 300 mg/kg pilocarpine (Sigma Aldrich, St. Louis, MO, United States). To reduce peripheral muscarinic effects, methylscopolamine (1 mg/kg) was intraperitoneally injected to the mice 30 min before pilocarpine injection. 5–10 min after pilocarpine administration, mice displayed facial and mouth movements, eye blinking, and head nodding. Mice were then developed discontinuous seizures about 30 min after pilocarpine injection and lasted up to hours. SE was defined as continuous tonic-clonic seizures following several discontinuous convulsive seizures. To analyze the spontaneous seizure activity, animals were subject to continuous video EEG recording with the video EEG monitoring system (Chengyi Inc., Chengdu, China). Electroencephalographic seizures were differentiated from background noise by the appearance of large-amplitude, high frequency activity, with progression of the spike frequency. The seizure intensity was assessed based on Racine scale: Stage 1, mouth and facial movements; Stage 2, head nodding; Stage 3, forelimb clonus; Stage 4, seizures characterized by rearing; Stage 5, seizures characterized by rearing and falling (Racine, 1972). The behavioral data captured by the synchronized video recording system were used to confirm EEG seizure activity.

### Brain Tissue Processing

For PCR and western blot experiments, the hippocampus were dissected, snap-frozen, and stored at −80°C until use. For immunocytochemistry experiment, the mice were euthanized by an intraperitoneal injection of an overdose of urethane and were transcardially perfused with 100 ml of saline (0.9% w/v NaCl), followed by 50 ml of 4% paraformaldehyde in 0.05 M sodium phosphate (pH = 7.4, containing 0.8% NaCl). The mouse brains were removed and post-fixed overnight in 4% paraformaldehyde, then were cryoprotected in 30% sucrose in PBS for 72 h. Serial coronal hippocampal sections with a thickness of 25 μm were cut using a cryostat (Leica Microsystems, Wetzlar, Germany) and every sixth section throughout the hippocampus were collected in PBS as free-floating sections and were stored at 4°C for future immunocytochemistry studies as we previously described (Zhu et al., 2016). For ADAM10 positive cells quantifications, one representative section from each of the five animals in different groups was picked. Eight regions of interest in the hippocampus from each section were then selected to count the ADAM10 positive cells.

### Bioinformatics

KEGG enrichment analysis of the association of miR-23a expression with enriched signaling pathways was performed by using mirPath (Version 3.0) software. Putative targets of miR-23a were predicted by TargetScan (Version 7.1)^1^, miRDB^2^, and DIANA MicroT^3^. GO analysis of predicted target genes was performed in terms of biological processes, which were identified by database from FunRich (Version 3.1.3). ADAM10 protein interaction network analysis was performed by STRING (Version 10.5)^4^.

### Luciferase Activity Assay

The mouse ADAM10 3′ UTR containing the putative miR-23a target site was PCR amplified by using the following primers: Forward 5′-GGCGGCTCGAGATCTGCAAATGATACCCTT AC-3′ and Reverse 5′-AATGCGGCCGCAAGCAGAAATCAGA CATCTA-3′. The DNA fragment was cloned into the Xho I and Not I sites at the end of the hRluc gene on the pmiR-RB-Report vector (RiboBio, Guangzhou, China). To construct the mutant ADAM10-3′-UTR vector, the miR-23a target site (GGAACCCA) within the ADAM10-3′-UTR was changed to CCTTGGGT by PCR mutagenesis with the following primers: Forward: 5′-ATGGAAAACCTTGGGTATTTTCTTATGAACAGAT-3′ and Reverse: 5′-AAGAAAATACCCAAGGTTTTCCATCCCACCC CCA-3′. For luciferase reporter assay, 293T cells were transfected with the wildtype ADAM10-3′-UTR and mutant ADAM10-3′-UTR vector, respectively. 48 h after transfection, the Dual-Glo Luciferase Assay System (Promega Corporation, Madison, WI, United States) were used to measure the luciferase activity. All experiments were performed in triplicate.

### Reverse Transcription and Quantitative Real-Time PCR

The dissected hippocampal tissues were homogenized and total RNA was extracted with Trizol reagent (Vazyme Biotech, Nanjing, China) according to the manufacturer’s instructions. Total mRNA (1 μg) was reverse transcribed using cDNA RT Kits (Vazyme Biotech, Nanjing, China). RNA and cDNA concentrations were measured using a spectrophotometer (OD-1000, Wuyi Technology, Nanjing, China). For quantitative real-time PCR (qRT-PCR), assays were performed with SYBR Green Master Mix (Vazyme Biotech, Nanjing, China) on a qRT-PCR System (Applied Biosystems, Foster City, CA, United States). The endogenous glyceraldehyde 3-phosphate dehydrogenase (GADPH) gene was used to normalize the level of the target mRNA in real time PCR. The primer sequence of ADAM10 and GADPH were as follows: ADAM10 Forward: 5′-CAACATCAAGGCAAACTATGCGA-3′; Reverse: 5′-CTTAGGTTCACTGTCCAAAGCGA-3′; GADPH: Forward: 5′-AAGGTCATCCCAGAGCTGAAC-3′; Reverse: 5′-TGAAGTCGCAGGAGACAACC-3′. For the analysis of miR-23a, miR-143, and miR-155, total RNA was isolated from hippocampus as described above. Comparative real-time PCR was performed using the Universal RT-PCR Master Mix (Vazyme Biotech, Nanjing, China). Specific primers and probes for mature miR-23a, miR-143, miR-155, and snRNA RNU6B were designed by RiboBio (Guangzhou, China). All reactions were run in triplicates. The amount of miR-23a, miR-143, and miR-155 were quantified by normalizing to snRNA RNU6B.

### Western Blotting

The dissected hippocampal tissues of the mice were homogenized in tissue lysis buffer (Beyotime Biotech, China). After being lysed for 15 min on ice, samples were centrifuged at 12,000 rpm for 15 min. The protein content in each supernatant fraction was determined using a BCA protein assay kit (Pierce, Rockford, IL, United States) and samples containing equivalent amounts of protein were applied to 12% acrylamide denaturing gels (SDS-PAGE). After electrophoresis, the proteins were transferred to nitrocellulose membranes (Amersham, Little Chalfont, United Kingdom) using a Bio-Rad mini-protein-III wet transfer unit (Hercules, CA, United States) overnight at 4°C. The membranes were then incubated with 5% non-fat milk in TBST (10 mmol/l Tris pH = 7.6, 150 mmol/l NaCl, 0.01%Tween-20) for 1 h at room temperature followed by three washes, then were incubated with mouse anti-ADAM10 (1:2000; Santa Cruz, TX, United States) and rabbit anti-β-actin (1:5000; Sigma-Aldrich, St. Louis, MO, United States) in TBST overnight at 4°C. After several washes with TBST buffer, the membranes were incubated for 1 h with HRP-linked secondary antibody (Boster Bioengineering, Wuhan, China) diluted 1:5,000, followed by four washes. The membranes were then processed with enhanced chemiluminescence (ECL) western blot detection reagents (Millipore, Billerica, MA, United States). Signals were digitally captured using a MicroChemi chemiluminescent image analysis system (DNR Bio-imaging Systems, Jerusalem, Israel). Blots were quantified using the Image J software (NIH, Bethesda, MD, United States).

### Statistical Analysis

All data are presented as the means ± SEM. Statistical significance was determined by using unpaired two-tailed Student’s *t*-test for two groups’ comparison and by using one-way ANOVA for multi-group comparisons. Tukey’s test was used for *posthoc* comparisons. The Pearson’s correlation coefficient analysis was used to analyze the correlations. Differences were considered to be significant for values of *p* < 0.05.

## Results

### miR-23a Expression Is Increased in the Hippocampus of Pilocarpine-Induced SE Mice

**FIGURE 1:**
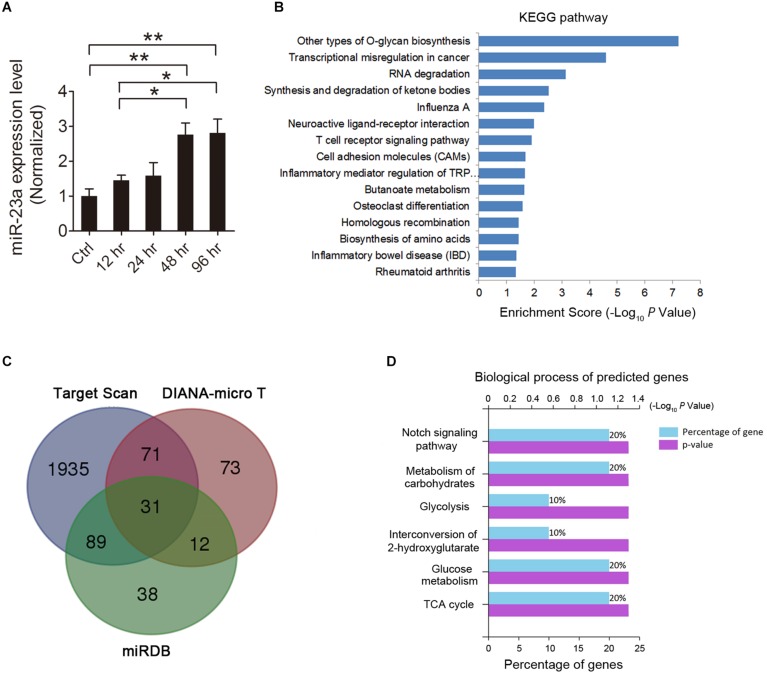
MicroRNA-23a (miR-23a) expression is increased in the hippocampus of temporal lobe epilepsy (TLE) mice. **(A)** Quantitative real-time PCR (qRT-PCR) analysis of miR-23a expression in the hippocampus of ctrl, 12, 24, 48, and 96 h post-status epilepticus (SE) mice (*F*_4,20_ = 7.07, *p* = 0.005, 48 h vs. ctrl; *p* = 0.004, 96 h vs. ctrl; *p* = 0.048, 48 vs. 12 h; *p* = 0.038, 96 vs. 12 h) (*n* = 5). **(B)** KEGG enrichment analysis of the association of miR-23a expression with enriched signaling pathways by using mirPath software. **(C)** Venn diagram showing the predicted target genes of miR-23a by three microRNA target prediction databases, including TargetScan, DIANA-MicroT, and miRDB. **(D)** GO analysis in terms of biological processes for the determination of the biological significance of the predicted miR-23a target intersection genes by the FunRich database. Values are mean ± SEM. *p < 0.05, **p < 0.01, one-way ANOVA.

MicroRNA represents a potential molecular mechanism for regulating gene expression in the pathological process of epilepsy. Recently, over a hundred miRNAs have been identified to be differentially expressed in the brains of epilepsy patients and animal epilepsy models (Henshall, 2014). Among these miRNAs, miR-23a was found to be up-regulated in epileptic brains of both humans and rats (Roncon et al., 2015), indicating its important role in the pathogenesis of epilepsy. To investigate the expression pattern of miR-23a in the hippocampus of pilocarpine-induced SE mice, we first examined the hippocampal miR-23a mRNA level by qRT-PCR at different time points after SE. Our results revealed that hippocampal miR-23a expression progressively increased from 12 to 96 h post-SE compared to those of control animals (Figure 1A). KEGG enrichment analysis demonstrated that miR-23a is enriched in the neuroactive receptor-ligand interaction pathway (Figure 1B), indicating its involvement in the regulation of neural activities. The potential targets of miR-23a were predicted by three miRNA target prediction databases, including TargetScan, DIANA-MicroT, and miRDB, which generated 31 intersectional miR-23a target genes (Figure 1C). These intersection target genes are *RNF185, ABHD2, DOCK11, SSMEM1, ERC2, ADHFE1, MTMR4, CNTN1, HAP1, PLEKHA6, PGK1, PLOD1, HSPB6, AGFG1, FYCO1, CSF1, TNFSF15, CS, COLGALT2, RBBP4, ADAM10, CEP57L1, TYW3, STXBP6, MICAL2, CHD4, IPO11, TBC1D10B, PKDCC, PYGM*, and *URM1.* Functional classification was then performed by GO analysis in terms of biological processes to determine the biological significance of these predicted miR-23a target intersection genes, which were identified using the database from FunRich. The analysis revealed that the intersectional predicted genes are enriched in the Notch signaling pathway (Figure 1D), which represents an important CNS molecular pathway in both physiological and pathological conditions.

### ADAM10 Is Validated as a Target Gene of miR-23a

**FIGURE 2:**
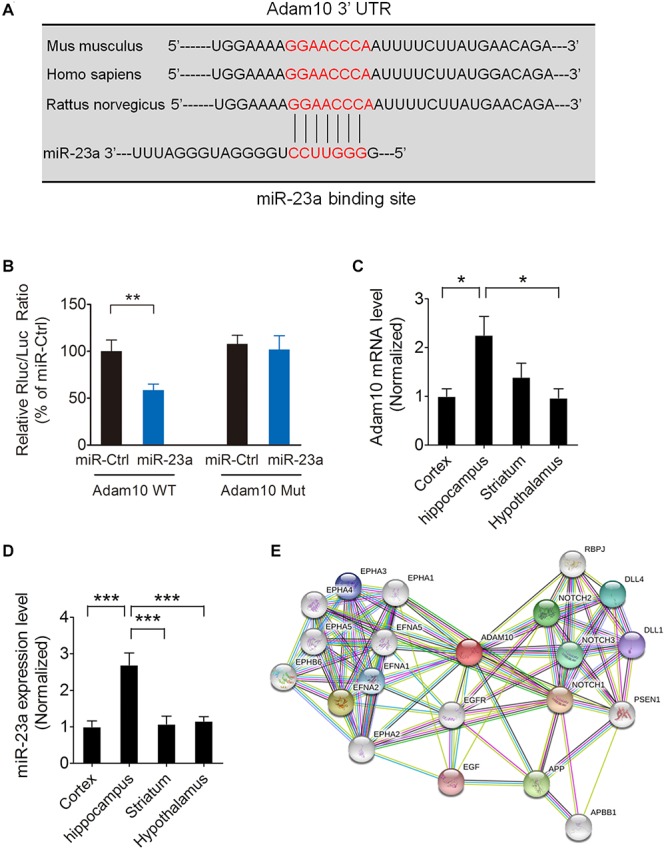
ADAM10is validated as a target gene of miR-23a. **(A)** The binding site for the miR-23a seed sequence in the ADAM10 mRNA 3′-UTR is highly conserved in mouse, human, and rat. **(B)** The relative luciferase activity of wild type or mutant ADAM10 3′UTR after cotransfection with a miR-23a mimic or a miR-23a mimic control in 293T cells (*p* = 0.0091). **(C)** qRT-PCR analysis of ADAM10 expression in the cortex, hippocampus, striatum, and hypothalamus (*F*_3,16_ = 4.96, *p* = 0.021, Hippocampus vs. Cortex; *p* = 0.018, Hippocampus vs. Hypothalamus) (*n* = 5). **(D)** qRT-PCR analysis of miR-23a expression in the cortex, hippocampus, striatum, and hypothalamus (*F*_3,16_ = 13.30, *p* < 0.001, Hippocampus vs. Cortex; *p* < 0.001, Hippocampus vs. striatum; *p* < 0.001, Hippocampus vs. Hypothalamus) (*n* = 5). **(E)** ADAM10 protein interaction network analysis performed by STRING software. Values are mean ± SEM. *p < 0.05, **p < 0.01, ^∗∗∗^*p* < 0.001, unpaired two-tailed Student’s *t*-test and one-way ANOVA.

To investigate whether ADAM10 is targeted by miR-23a, we used the TargetScan algorithm to predict whether miR-23a has a specific binding site at the 3′-untranslated region (3′-UTR) of the ADAM10 gene. A search of the database revealed that ADAM10 has a conserved miR-23a binding site within its 3′-UTR in most species (Figure 2A). To validate ADAM10 as a target of miR-23a, luciferase reporter vectors containing wild type or mutant ADAM10 3′-UTR, and a miR-23a mimic or mimic control were cotransfected into 293T cells. In the groups of wild type ADAM10, the luciferase activities were significantly repressed by miR-23a overexpression compared with the mimic control group. However, the luciferase activity of mutant ADAM10 3′-UTR was not different from miR-23a or mimic control group (Figure 2B), suggesting that ADAM10 is the target gene of miR-23a. Next, we assessed ADAM10 and miR-23a expression in different brain regions including the cortex, hippocampus, striatum, and hypothalamus by qRT-PCR. Our results showed that both of the ADAM10 and miR-23a expression levels are significantly higher in the hippocampus than in other brain regions such as the cortex and hypothalamus (Figures 2C,D), indicating that ADAM10 may have important functions in the hippocampus. ADAM10 protein interaction network analysis revealed a close relationship between ADAM10 and Notch (Figure 2E), which is in agreement with the GO analysis shown in Figure 1D. Taken together, these results indicate that pilocarpine-induced SE results in an increase in miR-23a expression in a time dependent manner. Furthermore, ADAM10 is validated as a target gene of miR-23a.

### ADAM10 Expression Is Decreased in the Hippocampus of Pilocarpine-Induced SE Mice

**FIGURE 3:**
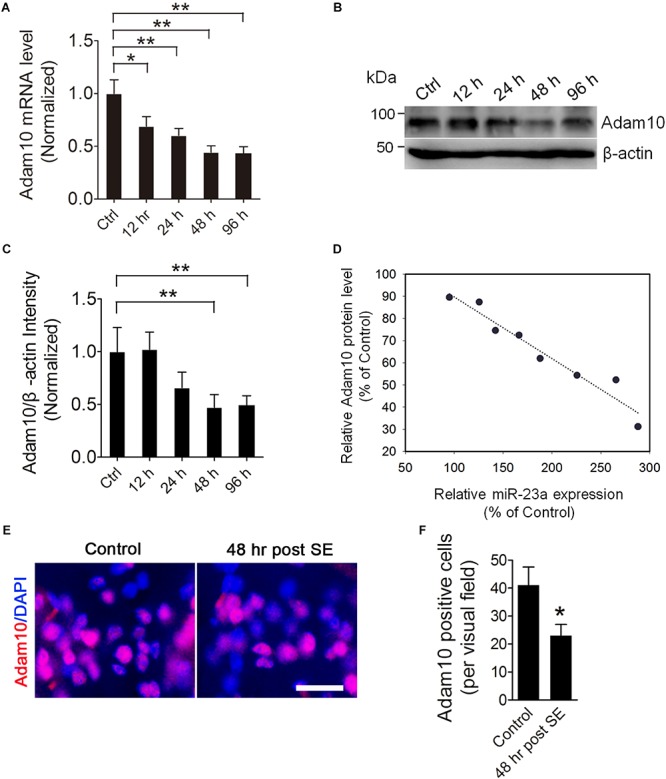
ADAM10 expression is decreased in the hippocampus of TLE mice. **(A)** qRT-PCR analysis of ADAM10 expression in the hippocampus of ctrl, 12, 24, 48, and 96 h post-SE mice (*F*_4,20_ = 7.19, *p* = 0.012, 12 h vs. ctrl; *p* = 0.007, 24 h vs. ctrl; *p* = 0.002, 48 h vs. ctrl; *p* = 0.001, 96 h vs. ctrl) (*n* = 5). **(B,C)** Western blotting analysis of ADAM10 protein levels in the hippocampus of ctrl, 12, 24, 48, and 96 h post-SE mice (*F*_4,20_ = 2.88, *p* = 0.006, 48 h vs. ctrl; *p* = 0.009, 96 h vs. ctrl) (*n* = 5). **(D)** Pearson correlation analysis of the correlation of relative ADAM10 protein level with miR-23a expression in the hippocampus of TLE mice (*r* = –0.97, *p* < 0.0001). **(E)** Representative images of ADAM10 immunostaining in the hilus region of hippocampal DG of vehicle ctrl and 48 h post-SE mice. **(F)** Bar graphs showing the quantification of ADAM10-positive cells in the hippocampus of the vehicle ctrl and 48 h post-SE mice, respectively (*p* = 0.035) (*n* = 5). Values are mean ± SEM. *p < 0.05, **p < 0.01, unpaired two-tailed Student’s *t*-test and one-way ANOVA. Scale bar = 50 μm.

To investigate the ADAM10 expression in the hippocampus of pilocarpine-induced SE mice, we first examined the ADAM10 mRNA level by qRT-PCR in the hippocampus of pilocarpine-induced SE mice. Our results revealed that ADAM10 mRNA levels were progressively reduced from 12 to 96 h post-SE (Figure 3A). In consistent with the qRT-PCR data, our western blot results showed that ADAM10 protein levels were also reduced from 12 to 96 h post-SE (Figures 3B,C). Immunohistochemistry results showed that ADAM10-positive cells in the hilus region of DG were significantly decreased at 48 h post-SE (Figures 3E,F), which further confirmed ADAM10 expression is decreased in the hippocampus of pilocarpine-induced SE mice. Moreover, a Pearson’s correlation coefficient analysis revealed a negative correlation between the ADAM10 protein level and miR-23a expression (Figure 3D). Taken together, these results indicate that pilocarpine-induced SE results in a decrease of ADAM10 expression in a time dependent manner.

### miR-23a Negatively Regulates Hippocampal ADAM10 Expression in Pilocarpine-Induced SE Mice

**FIGURE 4:**
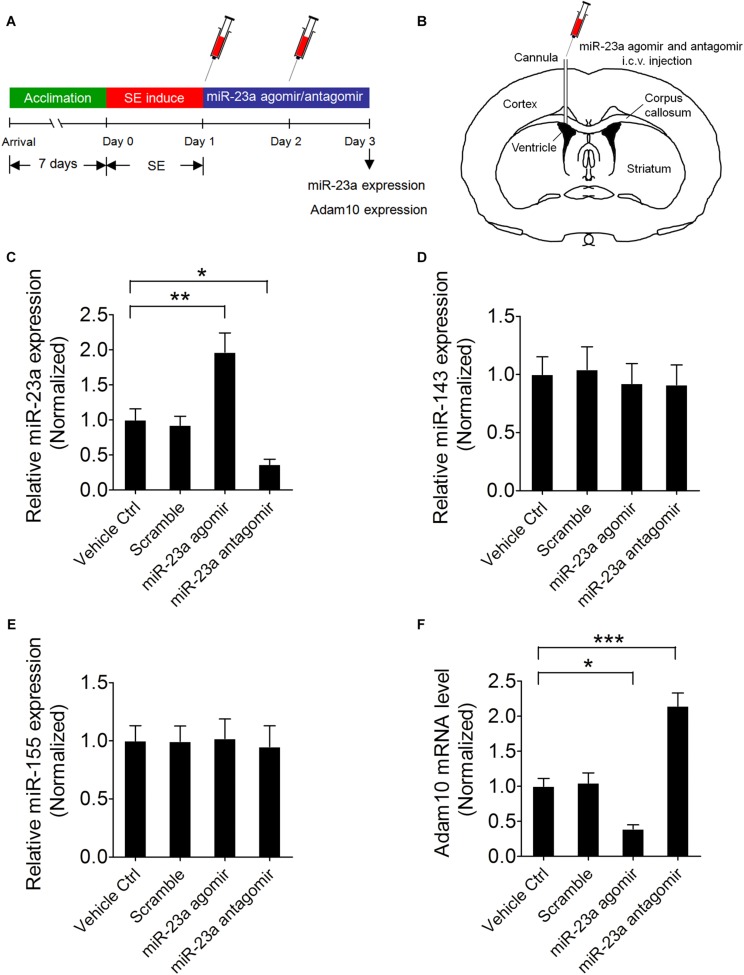
miR-23a down-regulates hippocampal ADAM10 mRNA level in TLE mice. **(A)** Schematic diagram of the experimental design. Mice were treated with a miR-23a agomir or miR-23a antagomir immediately after SE two times at 24 h interval and investigated miR-23a and ADAM10 expression levels 48 h after SE. **(B)** Schematic diagram of intracerebroventricular (i.c.v.) injection of miR-23a agomir or miR-23a antagomir into the lateral ventricle (LV). **(C–F)** qRT-PCR analysis of miR-23a (*F*_3,16_ = 14.66, *p* = 0.006, miR-23a agomir vs. vehicle ctrl; *p* = 0.048, miR-23a antagomir vs. vehicle ctrl), miR-143, miR-155, and ADAM10 (*F*_3,16_ = 29.87, *p* = 0.025, miR-23a agomir vs. vehicle ctrl; *p* < 0.001, miR-23a antagomir vs. vehicle ctrl) mRNA level in the hippocampus of vehicle ctrl, scramble, miR-23a agomir, and miR-23a antagomir-treated TLE mice (*n* = 5). Values are mean ± SEM. *p < 0.05, **p < 0.01, ***p < 0.001, one-way ANOVA.

**FIGURE 5:**
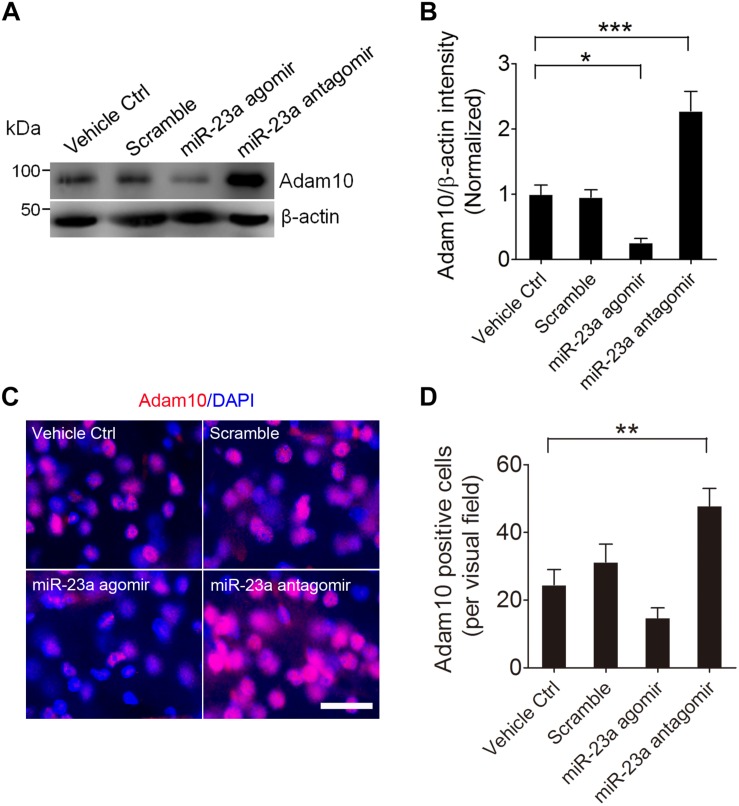
miR-23a down-regulates hippocampal ADAM10 protein level in TLE mice. **(A,B)** Western blotting analysis of ADAM10 protein levels in the hippocampus of vehicle ctrl, scramble, miR-23a agomir, and miR-23a antagomir-treated TLE mice. (*F*_3__,__16_ = 22.66, *p* = 0.042, miR-23a agomir vs. Vehicle Ctrl; *p* < 0.001, miR-23a antagomir vs. Vehicle Ctrl) (*n* = 5). **(C)** Representative images of the ADAM10 immunostaining in the hilus region of hippocampal DG of vehicle ctrl, scramble, miR-23a agomir, and miR-23a antagomir-treated TLE mice. **(D)** Bar graphs showing the quantification of ADAM10-positive cells in the hippocampus of the vehicle ctrl, scramble, miR-23a agomir, and miR-23a antagomir-treated TLE mice (*F*_3,24_ = 9.42, *p* = 0.007, miR-23a antagomir vs. vehicle ctrl) (*n* = 7). Values are mean ± SEM. *p < 0.05, **p < 0.01, ***p < 0.001, one-way ANOVA. Scale bar = 50 μm.

To further confirm that ADAM10 is regulated by miR-23a in the hippocampus of pilocarpine-induced SE mice, we treated the mice with miR-23a agomir or antagomir immediately after SE to up- or down-regulate miR-23a expression and investigated miR-23a and ADAM10 expression 48 h after SE (Figures 4A,B). Our qRT-PCR data revealed that miR-23a agomir treatment resulted in a significant increase in miR-23a levels, while miR-23a antagomir treatment resulted in a significant decrease in miR-23a levels in the hippocampus of pilocarpine-induced SE mice (Figure 4C). However, neither miR-23a agomir nor miR-23a antagomir altered levels of any other tested miRNAs such as miR-143 (Figure 4D) and miR-155 (Figure 4E). We proceeded next to study the effect of miR-23a on hippocampal ADAM10 mRNA levels in pilocarpine-induced SE mice. Our qRT-PCR data revealed that miR-23a agomir treatment resulted in a significant decrease in ADAM10 mRNA levels, while miR-23a antagomir treatment resulted in a significant increase in ADAM10 mRNA levels in the hippocampus of pilocarpine-induced SE mice (Figure 4F). Next, we examined hippocampal ADAM10 protein levels after up- or down-regulation of miR-23a by a miR-23a agomir or antagomir. In agreement with the mRNA results, our western blot analysis showed that miR-23a agomir treatment substantially decreased ADAM10 protein levels, while miR-23a antagomir substantially increased ADAM10 protein levels in the hippocampus of pilocarpine-induced SE mice (Figures 5A,B). Consistent with the western blot results, our immunochemistry results showed that miR-23a agomir treatment dramatically decreased the number of ADAM10-positive cells, while miR-23a antagomir treatment increased the number of ADAM10-positive cells in the hilus region of hippocampal DG of pilocarpine-induced SE mice (Figures 5C,D). Collectively, these data suggest that miR-23a negatively regulates ADAM10 expression in the hippocampus of TLE mice.

### miR-23a Regulates Seizure Activity Through Regulation of ADAM10

**FIGURE 6:**
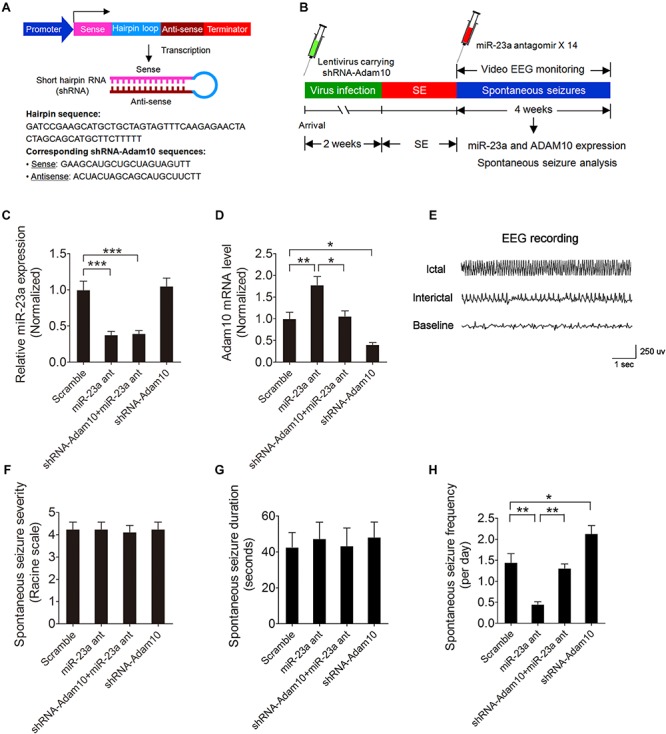
miR-23a regulation of seizure activity by targeting ADAM10. **(A)** Schematic diagram showing the lentiviral shRNA-ADAM10 expression vector system. The shRNA-ADAM10 is specific to the mouse ADAM10 mRNA. **(B)** Schematic diagram of experimental design. Mice were injected with lentivirus carrying ADAM10-shRNA into the hippocampal CA1 regions followed by SE induction. Immediately after SE, mice were then treated with miR-23a antagomir 14 times at 48 h intervals and subjected to continuous video EEG recording for 4 weeks. After the EEG recording was finished, the mice were sacrificed for hippocampal miR-23a and ADAM10 gene expression analysis. **(C,D)** qRT-PCR analysis of miR-23a (*F*_3__,1__6_ = 18.10, *p <* 0.001, miR-23a antagomir vs. scramble; *p* < 0.001, shRNA-ADAM10+miR-23a antagomir vs. scramble) and ADAM10 (*F*_3__,1__6_ = 16.32, *p =* 0.006, miR-23a antagomir vs. scramble; *p* = 0.011, shRNA-ADAM10+miR-23a antagomir vs. miR-23a antagomir; *p* = 0.037, shRNA-ADAM10 vs. scramble) expression level 4 weeks after Scramble, miR-23a antagomir, shRNA-ADAM10+miR-23a antagomir and shRNA-ADAM10 treatment in the hippocampus of TLE mice (*n* = 5). **(E)** A typical recording of the baseline, ictal and interictal EEG activity in the hippocampus. **(F–H)** Bar graphs showing the quantifications of average spontaneous seizure severity (*F*_3,28_ = 0.04, *p* = 0.99), spontaneous seizure duration (*F*_3,16_ = 0.10, *p* = 0.96), and spontaneous seizure frequency (*F*_3,__20_ = 19.58, *p* = 0.001, miR-23a antagomir vs. scramble; *p* = 0.005, shRNA-ADAM10+miR-23a antagomir vs. miR-23a antagomir; *p* = 0.026, shRNA-ADAM10 vs. scramble) in scramble, miR-23a antagomir, shRNA-ADAM10+miR-23a antagomir, and shRNA-ADAM10-treated TLE mice (*n* = 5–8). Values are mean ± SEM. *p < 0.05, **p < 0.01, ***p < 0.001, one-way ANOVA.

To determine whether miR-23a regulates spontaneous seizure activity in TLE mice and if so, whether this regulation is by targeting ADAM10 in the hippocampus of TLE mice, we first injected lentivirus carrying ADAM10-shRNA (Figure 6A) into the hippocampal CA1 regions of mice to knock down ADAM10 and then performed spontaneous seizure analysis of miR-23a antagomir-treated or vehicle control-treated SE mice continuously for 4 weeks (Figure 6B). To investigate the long-term effect of a miR-23a antagomir on miR-23a and ADAM10 expression, we tested miR-23a and ADAM10 expression levels 4 weeks after miR-23a antagomir treatment in the hippocampus of TLE mice. Our results showed that the down-regulation of miR-23a and up-regulation of ADAM10 by a miR-23a antagomir persisted for at least 4 weeks in the hippocampus of TLE mice (Figures 6C,D). We proceeded next to study the effect of miR-23a on spontaneous seizure activities in TLE mice. Following the episode of SE, we monitored the spontaneous recurrent seizures (SRS) by video EEG recording continuously for 4 weeks. An EEG recording showed a burst of large amplitude and high frequency spikes in the hippocampus of spontaneous seizure mice (Figure 6E). Our spontaneous seizure analysis showed that both spontaneous seizure severity and seizure duration did not change after miR-23a antagomir treatment (Figures 6F,G). However, spontaneous seizure frequency was significantly decreased after miR-23a antagomir treatment (Figure 6H). Interestingly, pretreatment with ADAM10-shRNA to knockdown ADAM10 abolished the effect of suppression of seizure activity by the miR-23a antagomir (Figure 6H). Notably, knockdown of ADAM10 by ADAM10-shRNA increased the spontaneous seizure frequency compared to scramble-treated TLE mice (Figure 6H), suggesting that miR-23a regulates spontaneous seizure activity by targeting ADAM10.

## Discussion

The present study reveals a critical role of miR-23a on epileptogenesis of TLE mice. We found that inhibition of miR-23a suppresses spontaneous recurrent seizures through up-regulation of ADAM10 in pilocarpine-induced SE mice. These data suggest a novel pathway functioning to suppress epileptogenesis, which may represent a therapeutic target in epilepsy.

MicroRNA-23a is a ncRNA located at chromosome 19 at location p13.13. Previous studies have reported that miR-23a is an important regulator in carcinogenesis, and aberrant miR-23a expression is associated with a variety of cancers (Gottardo et al., 2007; Mi et al., 2007; Saumet et al., 2009; Jahid et al., 2012). miR-23a has also been found to be involved in glutamine metabolism (Gao et al., 2009) and cardiac hypertrophy (Lin et al., 2009). Recent studies suggest that miR-23a also plays a pivotal role in the central nervous system (Lin et al., 2013; Sabirzhanov et al., 2014). In this study, KEGG enrichment analysis demonstrated that miR-23a is enriched in the neuroactive receptor-ligand interaction pathway, indicating its involvement in regulation of neural activities. Importantly, in this study, we found that miR-23a is abundantly distributed in hippocampus, an important structure involved in the pathophysiology of epilepsy.

The regulatory function of miR-23a in the hippocampus is dependent on its target genes. miRNA target prediction programs (TargetScan, DIANA-MicroT, and miRDB) were employed to identify the target genes of miR-23a, and 31 intersectional miR-23a target genes were generated. Among these genes, ADAM10 was markedly identified as one of the target genes, with the binding sites located at the 3′-UTR of ADAM10 by luciferase assay. We have repeated the assay for multiple times and the results are consistent. The ADAM10 gene was originally identified as an alpha-secretase in the processing of the APP, which is involved in AD. Recently, it has been reported that ADAM10 functions as a regulator of neuronal network activity and may play a crucial role in the pathological process of epilepsy (Prox et al., 2013; Kuhn et al., 2016). Here we found ADAM10 were abundantly expressed in the hippocampus, where many epileptic seizures begin. ADAM10 protein interaction network analysis revealed a close relationship between ADAM10 and Notch signaling, which represents an important CNS molecular pathway. Furthermore, we found that ADAM10 mRNA levels as well as the protein levels were progressively reduced from 12 to 96 h post-SE, inhibition of miR-23a increased hippocampal ADAM10 expression while an increase in miR-23a suppressed hippocampal ADAM10 expression, suggesting that ADAM10 is negatively regulated by miR-23a in the hippocampus of pilocarpine-induced SE mice. Transcriptional regulation occurs in a very short time window. The transcription factor binding usually takes seconds while the regulation process requires minutes. Post-transcriptional regulation of gene expression occurs between the process of transcription and translation of the gene. In particular, miRNA post-transcriptionally regulates gene expression through silencing or degradation of target gene mRNA, which usually requires hours to days. Therefore, in this study, we believe that ADAM10 is post-transcriptionally but not transcriptionally regulated by miR-23a.

An association between miR-23a and epilepsy has been suggested by recent studies (Song et al., 2011; Henshall, 2014; Roncon et al., 2015; Dombkowski et al., 2016). Here, we observed a progressive increase in miR-23a levels in the hippocampus of pilocarpine-induced SE mice. Our spontaneous seizure analysis revealed that inhibition of miR-23a decreased spontaneous seizure frequency. However, knockdown of ADAM10 abolished the effect of suppression of seizure activity by the miR-23a inhibitor. Collectively, these results suggested that inhibition of miR-23a suppressed spontaneous recurrent seizures through up-regulation of ADAM10 in pilocarpine-induced SE mice.

In conclusion, our data identify ADAM10 as a specific target gene of miR-23a, which contributes to epileptogenesis in TLE mice. Our results suggest that modulation of miR-23a-ADAM10 pathway could play a pivotal role in the development of epilepsy. The regulation of the miR-23a-ADAM10 pathway in the epileptic brain may offer a novel approach for the treatment of epilepsy.

## Ethics Statement

This study was carried out in accordance with the recommendations of Medical School of Southeast University. The protocol was approved by the Animal Care and Use Committee of Medical School of Southeast University. All efforts were made to minimize animal suffering and discomfort and to reduce the number of animals used.

## Author Contributions

XZ designed the research. XZ, YY, YL, RZ, WZ, QH, HL, and MAH performed the research. AZ provided the technical help. XZ analyzed the data and wrote the manuscript. All authors read and approved the final manuscript.

## Conflict of Interest Statement

The authors declare that the research was conducted in the absence of any commercial or financial relationships that could be construed as a potential conflict of interest.

## Footnotes

1 http://www.targetscan.org/vert_71/

2 http://www.mirdb.org

3 http://diana.imis.athena-innovation.gr/DianaTools/

4 http://string-db.org/
